# ZBTB7A as a novel vulnerability in neuroendocrine prostate cancer

**DOI:** 10.3389/fendo.2023.1093332

**Published:** 2023-03-29

**Authors:** Song Yi Bae, Hannah E. Bergom, Abderrahman Day, Joseph T. Greene, Zoi E. Sychev, Gabrianne Larson, Eva Corey, Stephen R. Plymate, Tanya S. Freedman, Justin H. Hwang, Justin M. Drake

**Affiliations:** ^1^ Department of Pharmacology, University of Minnesota-Twin Cities, Minneapolis, MN, United States; ^2^ Department of Medicine, University of Minnesota-Twin Cities, Minneapolis, MN, United States; ^3^ Division of Hematology, Oncology and Transplantation, University of Minnesota, Minneapolis, MN, United States; ^4^ Institute for Health Informatics, University of Minnesota, Minneapolis, MN, United States; ^5^ Department of Urology, University of Washington, Seattle, WA, United States; ^6^ Department of Medicine, Division of Gerontology and Geriatric Medicine, University of Washington, Seattle, WA, United States; ^7^ Geriatric Research, Education, and Clinical Center, Veterans Affairs (VA) Puget Sound Health Care System, Seattle, WA, United States; ^8^ Masonic Cancer Center, University of Minnesota-Twin Cities, Minneapolis, MN, United States; ^9^ Center for Immunology, University of Minnesota, Minneapolis, MN, United States; ^10^ Department of Urology, University of Minnesota-Twin Cities, Minneapolis, MN, United States

**Keywords:** ZBTB7A, neuroendocrine prostate cancer (NEPC), castration-resistant prostate cancer (CRPC), small-cell neuroendocrine (SCN), cancer dependency map (DepMap), RET receptor tyrosine kinase, gene network, cell cycle

## Abstract

Neuroendocrine prostate cancer (NEPC) is a highly aggressive subtype of prostate cancer. NEPC is characterized by the loss of androgen receptor (AR) signaling and transdifferentiation toward small-cell neuroendocrine (SCN) phenotypes, which results in resistance to AR-targeted therapy. NEPC resembles other SCN carcinomas clinically, histologically and in gene expression. Here, we leveraged SCN phenotype scores of various cancer cell lines and gene depletion screens from the Cancer Dependency Map (DepMap) to identify vulnerabilities in NEPC. We discovered ZBTB7A, a transcription factor, as a candidate promoting the progression of NEPC. Cancer cells with high SCN phenotype scores showed a strong dependency on RET kinase activity with a high correlation between RET and ZBTB7A dependencies in these cells. Utilizing informatic modeling of whole transcriptome sequencing data from patient samples, we identified distinct gene networking patterns of ZBTB7A in NEPC versus prostate adenocarcinoma. Specifically, we observed a robust association of ZBTB7A with genes promoting cell cycle progression, including apoptosis regulating genes. Silencing ZBTB7A in a NEPC cell line confirmed the dependency on ZBTB7A for cell growth *via* suppression of the G1/S transition in the cell cycle and induction of apoptosis. Collectively, our results highlight the oncogenic function of ZBTB7A in NEPC and emphasize the value of ZBTB7A as a promising therapeutic strategy for targeting NEPC tumors.

## 1 Introduction

Neuroendocrine prostate cancer (NEPC) is a sub-variant of aggressive prostate cancer that rarely arises *de novo*, but most frequently emerges after androgen receptor (AR)-targeted therapies for prostate adenocarcinoma (1). Second-generation AR pathway inhibitors, such as abiraterone acetate and enzalutamide, have improved the survival of patients with castration-resistant prostate cancer (CRPC). However, the increased use of these more potent and specific AR-targeted therapies has elevated the incidence of NEPC, and currently accounts for approximately one-fifth of metastatic CRPC (2–4). There are very limited therapeutic options, such as platinum-based chemotherapy, for NEPC patients and the median overall survival is short, only12-16 months (5, 6). In this regard, there is a great need to investigate the molecular characteristics of NEPC to develop promising targeted therapies that improve response rates and prolong overall survival of patients.

NEPC is commonly characterized by the loss of AR signaling in the process of transdifferentiation, which enables tumor cells to escape AR pathway inhibition reducing responsiveness to hormonal therapies. Other gene alterations that have been implicated in the development of NEPC include *RB1* deletion, *TP53* mutation, *AURKA* amplification, *EZH2* and *DLL3* overexpression as well as the activation of transcription factors (e.g. SOX2, ASCL1, NEUROD1, BRN2, and ONECUT2) (7–14). In our previous work, we reported that RET kinase overexpression strongly correlates with NEPC and plays a functional role in NEPC tumor progression (15). We hypothesized that combination therapies targeting RET with other agents may be an effective treatment strategy for NEPC. Thus, we aimed to find viability regulators of NEPC using RET as a key gene in NEPC.

Due to the limited NEPC models, we took advantage of the fact that small-cell neuroendocrine carcinomas (SCNC), including small-cell lung cancer (SCLC), and NEPC share similar genomic characteristics, such as *RB1* deletion and *TP53*, mutation independent of the tissue of origin (14, 16–18). A recent study by Balanis et al. rank ordered and categorized the gene expression-based prediction of small-cell neuroendocrine (SCN) phenotypes across tissues, utilizing normal, adenocarcinoma, and SCNC patient tumors. This allowed the characterization of tumors and cancer cell lines to be classified as either SCN-like or non-SCN-like using SCN scores. These scores allowed for a more robust interrogation of other vulnerabilities in NEPC using pan-cancer cell lines that mimic features of NEPC and reliance on RET for survival.

Through multiple informatics approaches, we identified the transcription factor ZBTB7A, as a potential regulator of NEPC proliferation and survival. ZBTB7A, also known as Pokemon (POK), LRF (lymphoma related factor) and FBI-1 (factor binding IST protein 1), consists of four zinc fingers and one BTB (Broad-Complex, Tramtrack and Bric a brac) domain, which allow it to bind to DNA and recruit various transcription factors (19). Besides its association with multiple physiological processes, such as cell proliferation, metabolism, adipogenesis and hematopoiesis, ZBTB7A plays oncogenic or onco-suppressive roles in several human cancers, depending on the tissue and cancer type (20–22). In prostate cancer, ZBTB7A is reported to induce cell proliferation in androgen insensitive prostate cancer cells (PC3) with increased ZBTB7A expression, while acting as a tumor suppressor in androgen sensitive prostate cancer cells (LNCaP) (23, 24). While some tumor suppressive mechanisms of ZBTB7A in specific prostate cancers have been reported (25–27), the oncogenic mechanism is unknown. As an oncogene in other cancers, ZBTB7A promoted tumorigenesis *via* suppression of the p14ARF-MDM2-p53 pathway and activation of NF-κB, TGF-β and the PI3K/AKT pathway leading to increased cell proliferation, metastasis, chemoresistance and inhibition of apoptosis (28–32). The accumulated evidence to date implicates ZBTB7A as a key player in cancer cell fate and thus, we sought to investigate whether ZBTB7A is critical for NEPC cell proliferation and survival.

## 2 Results

### 2.1 Small-cell neuroendocrine cancer cell lines exhibit dependency of RET and ZBTB7A

**Figure 1:**
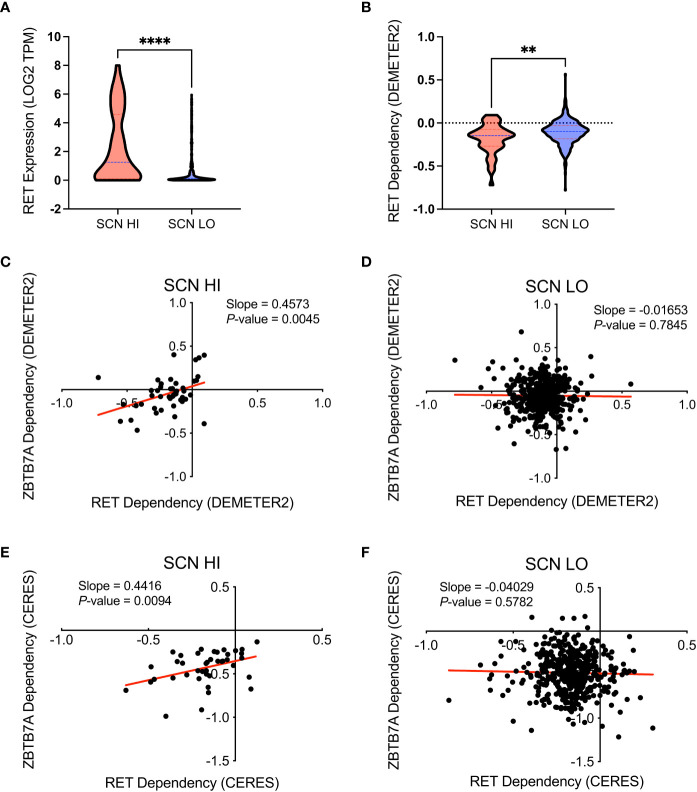
Functional co-dependency analyses identified small-cell neuroendocrine cancer cell lines to be dependent on RET and ZBTB7A. **(A)** Comparison of *RET* mRNA expression between cancer cell lines with SCN score ≥ 1.1 (SCN HI) and SCN score< 1.1 (SCN LO). *RET* mRNA expression values were obtained from Cancer Cell Line Encyclopedia Cancer. ****p<0.0001 by Mann-Whitney test. **(B)** Comparison of relative RET dependency scores (DEMETER2) between SCN HI and SCN LO cell lines. DEMETER2 scores taken from DepMap (DEMETER2 Data v6) reflect the impact of shRNA mediated RET knockdown on cell proliferation and the lower score indicates greater dependency on RET. **p<0.01 by Mann-Whitney test. **(C, D)** RET DEMETER2 scores of SCN HI **(C)** and SCN LO **(D)** cell lines were plotted against ZBTB7A DEMETER2 scores. **(E, F)** RET CERES scores of SCN HI **(E)** and SCN LO **(F)** were plotted against ZBTB7A CERES scores. CERES scores (DepMap Public 20Q4 v2) indicate the relative effect of gene perturbation by CRISPR/Cas9 on cell proliferation. **(C-F)** Each dot represents a cell line and the linear regression lines are in red.

Our previous work found that RET kinase activity was enriched in AR-low NEPC cell lines and its gene expression was upregulated in NE-positive patient tumors with a strong correlation with neuronal lineage markers (15). We sought to comprehensively evaluate the association of *RET* gene expression with SCN characteristics. We first classified cancer cell lines into two groups using the SCN scores that Balanis et al. established from the gene expression-and partial least-squares regression (PLSR)-based prediction of SCN phenotypes trained on the RNAseq data of lung adenocarcinoma and SCLC cell lines (16). Thus, we referenced the SCN scores of SCLC cell lines to set the cut-off value to divide the cell lines. We observed 83% (19/23) of SCLC cell lines above SCN score of 1.1 and 17% (4/23) of cell lines below 0.1 (**Supplementary Table 1**). Therefore, the 46 cell lines with SCN scores greater than 1.1 were categorized as high SCN-scored (SCN HI) cells and 445 cell lines with the scores less than 1.1 as low SCN-scored cells (SCN LO; **Supplementary Table 1**). As anticipated and shown in **Figure 1A**, *RET* mRNA expression, as based on the Cancer Cell Line Encyclopedia (https://depmap.org/portal/download), was significantly higher in SCN HI cell lines vs SCN LO cell lines. To further validate whether RET is an essential gene for cell proliferation or survival in SCN HI versus SCN LO cell lines, we performed a dependency analysis using genome-scale RNAi screens from the Cancer Dependency Map (DepMap; https://depmap.org/portal). The DEMETER2 score indicates the relative impact of gene suppression on cell viability of each cell line compared with other cell lines (33). Accordingly, the SCN HI cells exhibited greater relative dependency on RET than SCN LO cells as indicated by a lower DEMETER2 score (**Figure 1B**). This is consistent with our previous work demonstrating that NEPC cell lines have a stronger dependency on RET than prostate adenocarcinoma cell lines (15). These results suggest that RET, as a NEPC marker, can segregate the pan-cancer cell lines into SCN HI vs LO and SCN HI cell lines can be used as a surrogate for NEPC cell lines.

Having identified and validated that the SCN HI group of cell lines are NEPC-like, we aimed to identify novel genes as potential vulnerabilities in the SCN HI cell lines through an informatic approach. Using the DepMap data explorer, we specifically sought to examine genes that exhibited robust correlation with RET dependency in SCN HI cell lines. Among 16,810 genes that were available for co-dependency analyses against RET in the RNAi screening dataset, 695 genes significantly correlated with RET dependency (*P*< 0.05) in all of the 46 SCN HI cell lines. Of those genes, we focused on 6 transcription factors (SNAI3, HAND2, PBX1, CEBPG, ETV6 and ZBTB7A) that ranked in the top 100 genes positively correlated to RET dependency (**Supplementary Table 2**). After excluding genes (SNAI3, HAND2 and CEBPG) that were also co-dependencies of RET in SCN LO cell lines, we focused on ZBTB7A as a potential target in NEPC. A positive correlation was observed between the DEMETER2 scores of RET and ZBTB7A in the SCN HI group (Pearson’s *r* = 0.412, *P* = 0.005), while no correlation was observed in the SCN LO group (Pearson’s *r* = -0.013, *P* = 0.785 **Figures 1C, D**, **Table 1**).

**Table 1:** Correlation analysis of DEMETER2 and CERES in SCN HI and LO group of cell lines.

	Group of cell lines	Pearson	p-value	Spearman	p-value
DEMETER2	SCN HI	0.412	0.0045	0.386	0.0081
	SCN LO	-0.013	0.785	0.038	0.421
CERES	SCN HI	0.401	0.0094	0.417	0.0067
	SCN LO	-0.025	0.578	-0.011	0.817

To confirm these findings in an orthogonal approach, we also evaluated the CERES scores, a metric similar to DEMETER2 that measures the relative effect of CRISPR-Cas9 mediated depletion of a target gene on cell proliferation, of RET and ZBTB7A (34). Consistently, the CERES scores exhibited similar statistical trends to our findings with DEMETER2 (**Figures 1E, F**; **Table 1**; **Supplementary Table 3**). To account for potential outlier effects on Pearson correlation, we also adapted rank-based correlation approaches using Spearman correlation. The Spearman correlation coefficients from both DEMETER2 and CERES scores confirmed the positive relationship between RET and ZBTB7A dependency in SCN HI cell lines (**Table 1**). However, when *ZBTB7A* mRNA expression and dependency were observed alone in these same cell lines, the mRNA expression was higher in the SCN LO group and there was no difference in dependency between SCN LO vs SCN HI groups (**Supplementary Figure 1**). We further evaluated the co-expression of RET and ZBTB7A protein in LuCaP patient-derived xenograft (PDX) tumors. The majority of the NEPC tumors co-expressed RET and ZBTB7A protein, while the adenocarcinoma tumors primarily expressed only ZBTB7A (**Supplementary Figure 2**). This further suggests the potential co-dependency of NEPC patient tumors on RET and ZBTB7A. Taken together, these findings revealed that cell lines with higher SCN-like characteristics were dependent on both RET and ZBTB7A.

### 2.2 Gene networks associated with *ZBTB7A* are distinct in NEPC versus prostate adenocarcinoma

**Figure 2:**
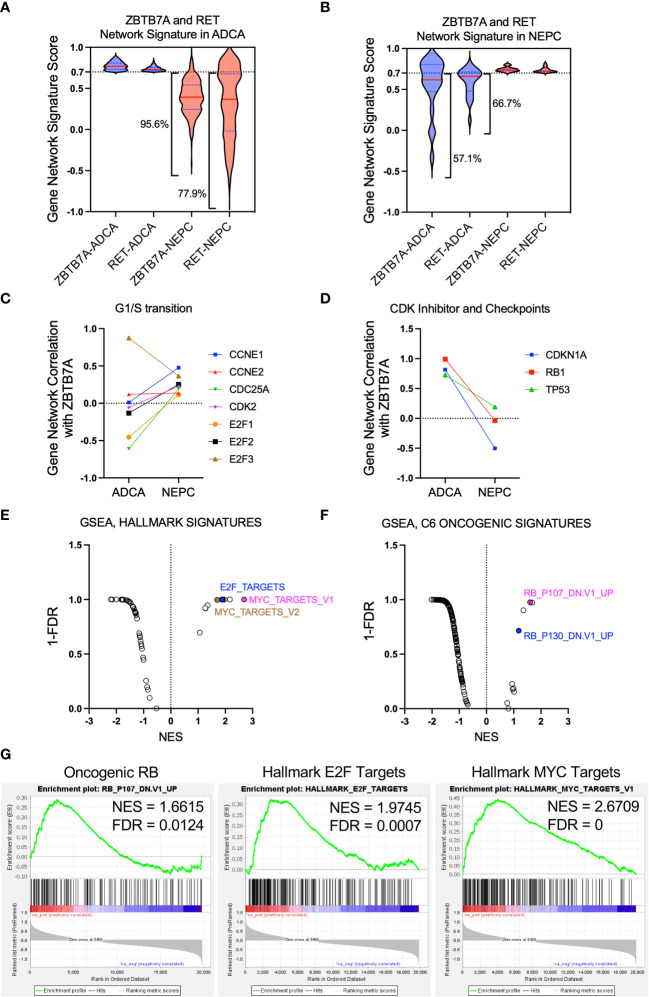
Gene network correlation analysis revealed distinct gene network associations with *ZBTB7A* in NEPC versus prostate adenocarcinoma. **(A, B)** Gene network signature from both *ZBTB7A* and *RET* in ADCA **(A)** or NEPC **(B)** was assessed in the other state and depicted using violin plots (see Methods). Gene network signature in each CRPC state was generated from the gene networks with correlation coefficients above 0.7 for both *ZBTB7A* and *RET*. The broad violin plots indicate the dissimilarity of correlation coefficients for a gene network signature. The percentages of gene network signatures below 0.7 are indicated on the plots. **(C)** Gene network correlation of genes related to G1/S transition with *ZBTB7A* in ADCA and NEPC. **(D)** Gene network correlation of CDK negative regulator (*CDKN1A*) and checkpoint (*RB1*, *TP53*) genes with *ZBTB7A* in ADCA and NEPC. **(E, F)** GSEA analysis of *ZBTB7A* gene profiles based on NEPC and ADCA tumors from Beltran et al., 2016 (see Methods). Each dot represents a gene signature. Among the signatures enriched in *ZBTB7A* gene profile of NEPC, the cell cycle progression related ones are labeled. For the full list of signatures, see **Supplementary Table 4** **(G)** Individual enrichment profiles are shown.

The discrepancy of RET and ZBTB7A dependency in SCN HI versus SCN LO cell lines (**Figure 1**) indicated that these genes may exhibit distinct gene interactions in NEPC compared to prostate adenocarcinoma. Using whole transcriptome sequencing (WTS) data from tumors of patients with metastatic CRPC (1), we explored the gene networks of *ZBTB7A* to elucidate gene behavior and gene associations in patient samples. Here, we define gene network as the identification of consistent patterns of gene expression across all patients and in relation to all genes within the transcriptome. The Beltran et al., 2016 dataset includes samples with histologic features of either castration-resistant prostate adenocarcinoma (ADCA, n=33) or neuroendocrine cancers (NEPC, n=14) and was separated into these two subtypes before analysis. We first constructed gene networks of all sequenced genes based on the relative association of all gene pairs across all samples from ADCA (genes = 19,957) and NEPC (genes = 19,835) (see Methods). The gene networks of *ZBTB7A* and *RET* which were determined by correlating each gene profile to that of all other genes, were quantitatively compared across the two cohorts. Gene networks of 19,769 genes were observed in both cohorts. From these gene networks, genes with robust correlations (cut-off 0.7) were defined as a gene network signature within a specific context. As shown in **Figure 2A**, the overlapped gene network signatures of *RET* and *ZBTB7A* in ADCA had reduced association in NEPC as depicted by the broader distribution of violin plots. Among the overlapped gene network signatures in ADCA, 77.9% of RET and 95.6% of ZBTB7A gene networks in NEPC were below the cut-off 0.7. On the contrary, gene networks highly associated with both *ZBTB7A* and *RET* in NEPC exhibited a lesser degree of congruence in the ADCA samples as 57.1% and 66.7% of those were below 0.7 cut-off for the ZBTB7A and RET gene networks, respectively, (**Figure 2B**). Overall, these results suggest that a subset of gene networks in both *RET* and *ZBTB7A* are shared with one another but that these gene networks are dynamic and distinct across these two CRPC subtypes. This reflects the difference in dependency of ZBTB7A and RET when comparing SCN HI to SCN LO cell lines we demonstrated in **Figure 1**.

Several studies have shown that ZBTB7A promotes cell proliferation by inducing cell cycle progression (35–37). Moreover, ZBTB7A is known to stimulate the promoter activity of E2F target genes, including *CCNE1* (encoding Cyclin E), where E2F family of transcription factors are well known regulators of S-phase entry (38). To further understand these roles of ZBTB7A in NEPC versus ADCA, we examined gene networks of genes that are involved in G1/S transition of the cell cycle including E2F target genes (39). As shown in **Figure 2C**, the gene networks of transcriptional activating E2F genes, *E2F1* and *E2F2*, and the E2F target genes, including *CCNE1*, *CDC25A* and *CDK2*, negatively correlated with the *ZBTB7A* gene network in ADCA samples. On the contrary, these same genes showed an overall positive correlation with the *ZBTB7A* gene network in NEPC samples. These context-specific positive associations with ZBTB7A in NEPC suggest that ZBTB7A has a similar behavior with genes that promote G1/S transition in NEPC but not in ADCA.

Another oncogenic function of ZBTB7A is to block the negative regulation of the cell cycle by competing with p53 to repress the transcription of the CDK negative regulator *CDKN1A* (encoding p21) and by transcriptionally suppressing *RB1* (40, 41). To further understand this relationship in NEPC versus ADCA, we examined the gene network correlation between *ZBTB7A* and genes that negatively regulate cell cycle such as *CDKN1A*, *RB1* and *TP53*. All three gene networks were highly associated with *ZBTB7A* in ADCA samples as indicated by their correlation coefficient of close to 1.0 (**Figure 2D**). However, this positive correlation was not conserved in NEPC samples, indicating that the behavior of ZBTB7A in suppressing cell cycle progression may vary depending on the subtype of CRPC. Overall, these findings suggest the behavior of ZBTB7A in NEPC favors cell cycle progression, such as promoting gene function with regard to G1/S transition and suppression of genes negatively regulating cell cycle.

To confirm our gene network analyses, we sought to examine the association of ZBTB7A with known cell cycle genes and pathways. We performed GSEA (42) to determine transcriptional programs robust in NEPC versus ADCA based on their differences in *ZBTB7A*-gene associations. By examining 50 hallmark signatures and 187 oncogenic signatures from MsigDB (43), we found enrichment of several gene sets that reflected activation of oncogenic and hallmark signatures in NEPC (**Figures 2E–G**; **Supplementary Table 4**). Specifically, we identified gene and hallmark signatures involved in cell cycle progression, including genes upregulated by *RB1* loss, and target genes of E2F and MYC. Taken together, the gene network correlation analysis and GSEA data suggest that in NEPC, ZBTB7A is robustly associated with genes and signaling programs that control cell cycle.

### 2.3 Silencing *ZBTB7A* inhibits cell proliferation in RET dependent NEPC cells *via* cell cycle regulation

**Figure 3:**
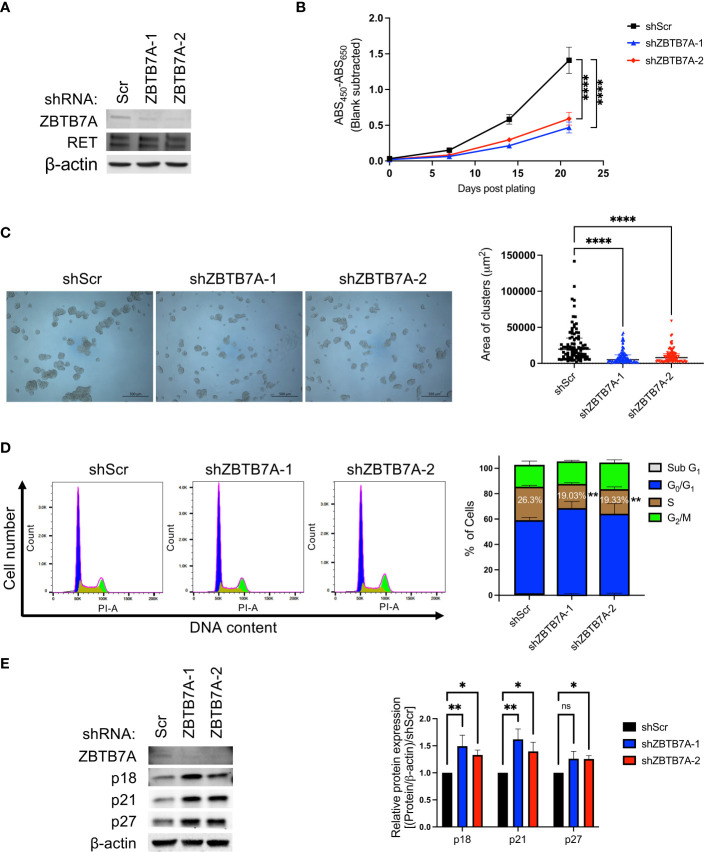
*ZBTB7A* knockdown reduced cell proliferation of NCI-H660 cells by blocking progression through the cell cycle. **(A)** ZBTB7A and RET protein expression in NCI-H660 cells stably transduced with scrambled (Scr) or two anti-*ZBTB7A* shRNAs. β-actin was used as a loading control. **(B)** Proliferation of stably transduced cells was measured every 7 days for 3 weeks *via* WST assay. Data are representative of three biological replicates. Error bars represent the SD of six technical replicates. Significance on Day 21 was assessed using one-way ANOVA. ****p<0.0001. **(C)** Representative images of each cell line in B at Day 21. Area of 90 cell clusters for each cell line (15 cell clusters/technical replicate, *n* = 6) were measured using Zen lite software and shown as a scatter plot with median and interquartile range. ****p<0.0001 by Kruskal-Wallis test. **(D)** Cell cycle analysis in NCI-H660 shScr and *ZBTB7A* knockdown cells using flow cytometry. Representative histograms showing cell cycle distribution of each cell line cultured for 48 hours after synchronization. The percentage of cell population in each cell cycle phase was calculated using FlowJo software. The stacked bars represent the mean from three biological replicates, and the error bars are SD. The statistical significance of percentage of cells in S phase was assessed against NCI-H660 shScr cells by one-way ANOVA. **p<0.01. **(E)** Expression of ZBTB7A and CDK-inhibitory proteins in NCI-H660 shScr and *ZBTB7A* knockdown cells. Protein expression levels were quantified by densitometry using Image J software. Bars represent the mean from five biological replicates, and error bars are SD. *ns*, non-significant, *p<0.05, and **p<0.01 by Kruskal-Wallis test.

Since our informatic analyses of genome-wide RNAi and CRISPR loss-of-function screens were performed in pan-cancer cell lines, we validated our findings using NCI-H660, a NEPC cell line (15). We generated stable *ZBTB7A* knockdown by transducing cells with either of two shRNAs targeting *ZBTB7A*. Downregulation of ZBTB7A protein in the stably transduced cells were confirmed by western blot (**Figure 3A**). Silencing *ZBTB7A* did not alter the expression of RET protein in either cell line, indicating that ZBTB7A, as a transcription factor, did not affect the transcriptional control of *RET*. A reduction in cellular proliferation was observed in the *ZBTB7A* knockdown cells compared to the scrambled shRNA transduced cells (shScr) (**Figure 3B**). At day 21, cell growth was decreased by 66.7% and 58.0% in shZBTB7A-1 and shZBTB7A-2 cells, respectively. Consistent with this finding, the size of *ZBTB7A* knockdown cell clusters were significantly smaller than that of the scrambled shRNA cells on day 21 (**Figure 3C**; **Supplementary Figure 3**).

Previous studies reported that silencing of *ZBTB7A* induces G1 cell cycle arrest and a consequent reduction in the S phase population in various human cancer cell lines, implicating ZBTB7A in cellular processes such as proliferation, senescence and apoptosis (35–37, 44). In addition, our initial informatics analyses (**Figures 2C–G**) further suggested the involvement of ZBTB7A in cell cycle control in NEPC patient tumors. Thus, we investigated whether silencing *ZBTB7A* alters the cell cycle of NCI-H660 cells. Cell cycle analysis revealed a significant reduction of S phase cell entry in shZBTB7A-1 and -2 cells compared to shScr cells (**Figure 3D**). The relative reduction of cell entry into the S phase was 28% and 27% for shZBTB7A-1 and -2 cells, respectively, compared to shScr cells. Moreover, ZBTB7A has been reported to suppress the expression of CDK negative regulators, which bind to and inactivate cyclin-CDK complexes, preventing cell cycle progression (32, 45). The expression of CDK-inhibitory proteins (p18, p21 and p27) were significantly induced in *ZBTB7A* knockdown cell lines compared to the shScr cell line (**Figure 3E**). Taken together, these results in NCI-H660 cells confirmed our findings from the initial informatics analyses using patient samples that ZBTB7A regulates cell cycle in NEPC and this promotes the proliferation of NEPC cells.

### 2.4 Silencing *RET* in *ZBTB7A* knockdown NEPC cells further suppresses cell cycle progression

**Figure 4:**
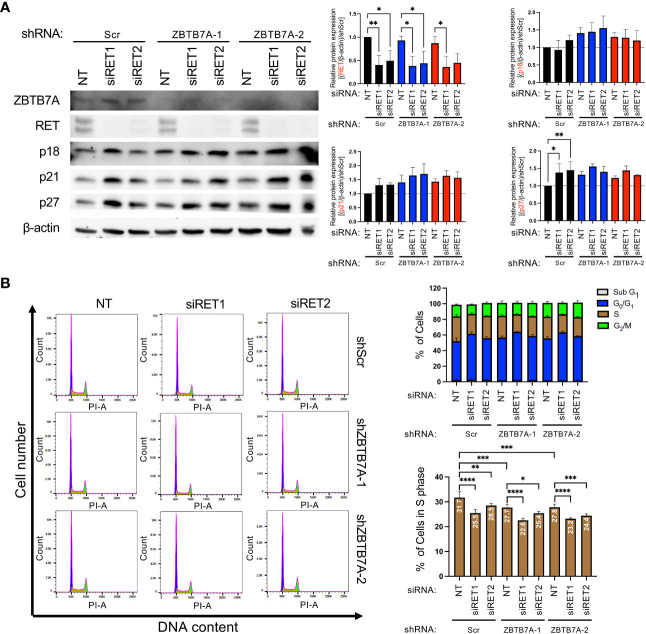
Silencing *RET* in NCI-H660 *ZBTB7A* knockdown cells further reduced cell entry into S phase. **(A)** Protein expression of ZBTB7A, RET and CDK negative regulators in NCI-H660 shScr and *ZBTB7A* knockdown cells after transient transfection with non-targeting (NT) or two unique anti-*RET* siRNAs for 96 hours, including serum-starvation during the first 24 hours of transfection. Protein expression levels were quantified by densitometry using Image J. Bars represent the mean from five biological replicates and error bars are SD. *p<0.05, and **p<0.01 by one-way ANOVA. All the unmarked pairs are statistically non-significant. **(B)** Representative histograms of cell cycle analysis by flow cytometry in NCI-H660 shScr and *ZBTB7A* knockdown cells after transient transfection with indicated siRNAs following the condition described in **(A)**. Cell cycle distributions were analyzed using FlowJo. Bars represent the mean percentage of cells in indicated cell cycle phase from four biological replicates and error bars are SD. *p<0.05, **p<0.01, ***p<0.001, and ****p<0.0001 by one-way ANOVA.

According to the co-dependency analyses (**Figures 1C–F**), SCN HI cell lines were dependent on both ZBTB7A and RET for cell survival. Therefore, we investigated whether silencing *RET* can further inhibit cell cycle progression in *ZBTB7A* knockdown NEPC cells. Two independent siRNAs targeting *RET* were confirmed to downregulate RET protein expression in the cell lines when compared to the non-targeting (NT) siRNA control (**Figure 4A**). In addition, we assessed p18, p21 and p27 protein expression in the cells with *ZBTB7A* and *RET* double knockdown. Although not statistically significant, we observed a slight increase in the expression of three CDK-inhibitory proteins in the double knockdown cells (**Figure 4A**). We therefore assessed the cell cycle distribution of these cell lines. In the shScr cell line, the suppression of RET alone reduced cell entry into S phase compared to the NT siRNA transfection (**Figure 4B**). The relative reduction of the S-phase cell population was 20% and 10% for siRET1 and siRET2 cells, respectively, when compared to shScr cells. Furthermore, we observed a significant decrease in the percentage of cells in S phase by silencing *RET* in *ZBTB7A* knockdown cell lines. The shZBTB7A cell lines had a 12-13% reduction in S phase relative to the shScr cell lines, and the S-phase populations were further reduced by 8-18% in double knockdown cells compared to the corresponding shZBTB7A cell lines. These findings suggest that RET and ZBTB7A have independent abilities to regulate S phase entry and the depletion of both genes leads to an additive effect on inhibiting cell cycle progression.

### 2.5 *ZBTB7A* knockdown induces apoptosis in NEPC cells

**Figure 5:**
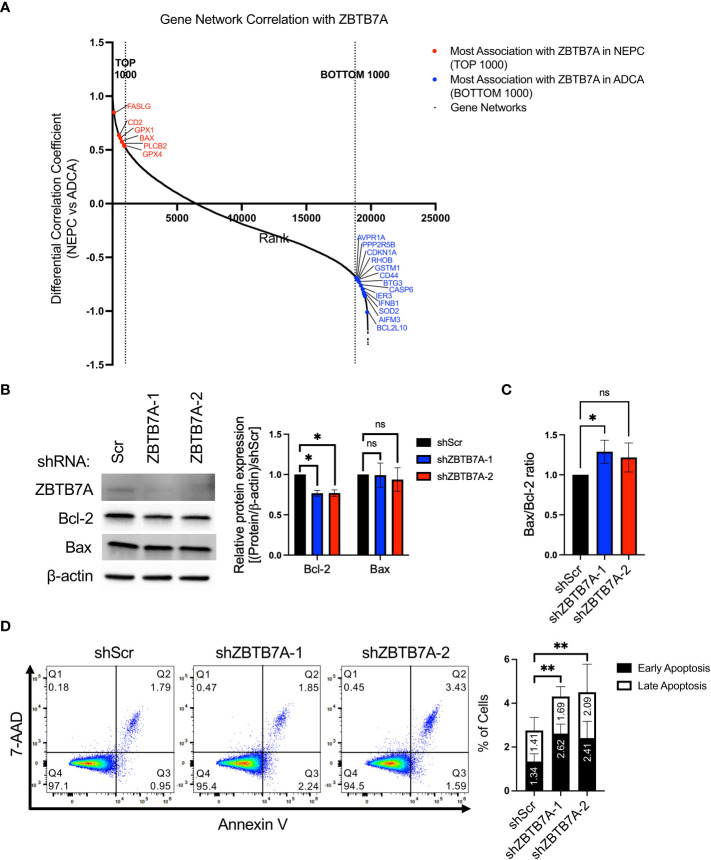
Gene network analysis and *ZBTB7A* perturbation revealed the association of ZBTB7A in suppressing apoptosis in NEPC. **(A)** Gene networks of apoptosis related genes are associated with *ZBTB7A* gene network in NEPC and ADCA. The gene network correlation coefficients of 19,769 genes present in both ADCA and NEPC patients were used. The gene networks associated with *ZBTB7A* were plotted in the rank order based on the differential gene network correlation coefficient values (see Methods). High differential correlation coefficient value indicates that a gene network is more associated with *ZBTB7A* in NEPC, while low value implies more association with *ZBTB7A* in ADCA. Red and blue dots represent the most associated gene networks of apoptosis related genes with *ZBTB7A* in NEPC and ADCA, respectively. **(B)** ZBTB7A, Bcl-2 and Bax protein expression in NCI-H660 shScr and *ZBTB7A* knockdown cells. Bcl-2 and Bax protein levels were quantified by densitometry using Image J. Bars represent the mean from four biological replicates, and error bars are SD. *ns*, non-significant, *p<0.05 by Kruskal-Wallis test. **(C)** Bax/Bcl-2 ratio was calculated using their quantified protein expression from each biological replicate in **(B)**. Bars represent the mean of relative differences to the ratio of shScr cells from four biological replicates, and error bars are SD. *ns*, non-significant, *p<0.05 by Kruskal-Wallis test. **(D)** Apoptosis assay in NCI-H660 shScr and *ZBTB7A* knockdown cells by flow cytometry using Annexin V-FITC/7-AAD double staining. Representative scatter plots of 7-AAD versus Annexin V. The stacked bars represent the mean percentage of early and late apoptotic cells from three biological replicates and error bars are SD. The total percentage of apoptotic cells was assessed using one-way ANOVA. **p<0.01.

Several studies have shown that silencing *ZBTB7A* induces apoptosis in cancer cells further sensitizing the cells to chemotherapies (36, 37, 44). To investigate whether ZBTB7A differentially regulates apoptosis in NEPC versus ADCA tumors, we first examined the association of the *ZBTB7A* network with genes related to apoptosis. To do so, we calculated the differential gene network correlation coefficients by subtracting the correlation coefficients for all genes with *ZBTB7A* in ADCA patient samples from that in NEPC (**Supplementary Table 5**). This revealed gene networks that have the largest change in association with *ZBTB7A* gene networks from NEPC to ADCA. Among the 19,769 gene networks common to NEPC and ADCA samples, we observed that multiple apoptosis related genes displayed a changed gene networking pattern with *ZBTB7A* in NEPC compared to ADCA (**Figure 5A**). While we found 6 genes involved in apoptosis regulation within the top 1,000 genes that were more associated with *ZBTB7A* in NEPC, 13 other apoptosis related genes ranked within the bottom 1,000 that were more associated with *ZBTB7A* in ADCA. This implies that ZBTB7A may interact with apoptosis regulating genes in both NEPC and ADCA tumors leading to either inducing or suppressing apoptosis.

To further investigate the relationship between ZBTB7A and apoptosis, we examined apoptosis-regulating proteins and performed an assay to evaluate early and late stages of apoptosis. While expression of the pro-apoptotic protein Bax remained unchanged across the cell lines, the anti-apoptotic protein Bcl-2 expression was significantly decreased in *ZBTB7A* knockdown cell lines compared to shScr cell line (**Figure 5B**). We measured the ratio of Bax/Bcl-2, which indicates cells undergo apoptosis when the ratio increases (46). We also observed a modest increase in the Bax/Bcl-2 ratio in ZBTB7A knockdown cells compared to shScr cells (**Figure 5C**). There was also a statistically significant increase in the percentage of apoptotic cells after silencing *ZBTB7A*, as indicated by Annexin V-positive or Annexin V/7-AAD double positive staining (**Figure 5D**). The relative percentage increases were 57% and 64% for shZBTB7A-1 and -2, versus shScr cells. Taken together, these results confirmed the proposed association of apoptosis with ZBTB7A in NEPC from the informatic analysis and further suggested that ZBTB7A suppresses apoptosis, which can consequently lead to increased cell proliferation.

## 3 Discussion

The increased occurrence of treatment-emergent NEPC and the lack of effective treatment options available for this disease emphasize the importance of identifying, testing, or developing new therapies for patients with NEPC. Utilizing SCN scores and gene dependency scores, we demonstrated that SCN-like cancer cell lines resemble NEPC cell lines in terms of RET expression and dependency. This allowed us to identify ZBTB7A as a key factor in cancer cell survival from the broader set of NEPC-like cancer cell lines. We also showed that ZBTB7A has similar gene behavior to those promoting G1/S transition in NEPC, but not in ADCA. By perturbing *ZBTB7A* expression, we observed a decrease in cell proliferation and an increase in apoptosis suggesting that the gene is required for the progression of NEPC. Transient silencing of *RET* in the *ZBTB7A* knockdown cells was additive on inhibiting S-phase cell entry. These findings indicate that ZBTB7A plays an essential role in NEPC progression and thus may serve as a promising therapeutic target, alone or in combination with RET inhibitors, in NEPC treatment.

Assessing the genetic dependencies in cancer presents new opportunities for identifying cancer vulnerabilities and potential targets for drug discovery (47). Recently, the large-scale datasets from lentiviral-based RNAi or CRISPR/Cas9 screens consisting of over 17,000 genes across more than 700 cancer cell lines in approximately 30 cancer lineages became available through DepMap. DepMap further provides analytic tools that allows comparison of the relative dependency of cell lines to each gene and assessment of correlations between gene dependencies (33, 34). While this new approach allowed us to confirm the difference in RET dependency between SCN HI and LO cell lines, the ZBTB7A dependency scores alone failed to segregate cell lines into two groups, probably due to its counter roles in cancer. However, the co-dependency with RET revealed the association of ZBTB7A in SCN HI cells, suggesting that co-dependency analysis with RET, a possible emerging NEPC target, can distinguish the roles of ZBTB7A in different prostate cancer subtypes.

In this study, we demonstrated that gene network analysis can be utilized as a robust tool for comparing the behavior of specific genes in different cancer subtypes and evaluating potential functional roles in cancers using clinical datasets. Gene network analyses revealed distinct correlations of gene networks with both ZBTB7A and RET in NEPC versus ADCA. This allowed for further exploration into the differential gene networking patterns of both ZBTB7A and RET in each subtype. Previous studies described that ZBTB7A can exhibit opposing functions as a transcription factor, acting as a tumor suppressor or oncogene in different tissues and cancer types. Because gene network analysis constructs context-specific and informatically driven phenotypes, this approach allowed us to interrogate the potential biological functions of ZBTB7A in NEPC. This approach in investigating genes with bi-functional roles is an invaluable tool to increase understanding of cancer biology (48).

The E2F/RB module is a key transcriptional regulator of many genes required for G1/S transition (49). Unfortunately, *RB1* gene alterations that inactivates its function to regulate E2Fs are commonly found in a vast majority of human tumors, including NEPC (1, 50). Therefore, it is important to find alternative modes that control E2F signaling and cell cycle progression. We showed that silencing *ZBTB7A* in NEPC cells decreases the S phase cell population potentially in part *via* the de-repression of *RB1*, *TP53*, and *CDKN1A* transcription and *via* repression of E2F-dependent gene transcription according to previous studies. Our results from the gene network analysis and GSEA reflected these functions of ZBTB7A in NEPC for all genes except the *E2F3* gene network. *E2F3*, one of the activator E2F genes, exhibited a similarly strong interaction in both CRPC subtypes. *E2F3* encodes for the two protein isoforms, E2F3a and E2F3b, where E2F3b functions as a repressor in some types of quiescent cells (51). However, the overexpression of *E2F3* is reported to correlate with poor overall survival in prostate cancer patients suggesting that the network correlation of this gene may be independent of any tumor phenotype (52). The increased expression of CDK-inhibitory proteins by silencing ZBTB7A in NEPC cells further confirmed the potential role of ZBTB7A on cell cycle control. While the precise mechanism in regulating the promotor activity of cell cycle genes by ZBTB7A remains to be elucidated, our results suggest that the involvement of ZBTB7A in controlling E2F activity and cell cycle in NEPC is promising.

From a clinical perspective, NEPC patients are typically treated with platinum-based chemotherapies, which cause cell death by inducing apoptosis after DNA damage. However, a majority of NEPC tumors were reported to lack apoptotic activity (53), and this could explain the limited therapeutic response. We demonstrated that silencing *ZBTB7A* increases the apoptotic cell population in NEPC cells and downregulates protein expression of Bcl-2, which plays a role in apoptosis inhibition. Therefore, we anticipate that suppressing ZBTB7A may result in NEPC tumors to be more susceptible to apoptosis, and this may improve patient responses to apoptosis-inducing chemotherapies.

Although we nominate ZBTB7A as a target in NEPC treatment, there are no available ZBTB7A pharmacologic agents to date. Since we have demonstrated that ZBTB7A induces G1/S transition, the inhibitors that modulate the transcription or activity of E2F, such as BET and CDK4/6 inhibitors, may be alternative therapeutic strategies to targeting ZBTB7A. In NEPC, the binding of BRD4, a member of bromodomain and extraterminal (BET) protein family, at the *E2F1* promoter results in upregulation of *E2F1* (54). The pan-BET inhibitors, such as JQ1 and ZEN-3694, and a pan-BET degrader, ARV-771, are reported to effectively inhibit cell survival, E2F1 function, and expression of NEPC lineage plasticity genes. CDK4/6 inhibitors (e.g. palbociclib, ribociclib, abemaciclib) block the G1/S transition by suppressing the kinase activity of the CDK/cyclin complex, which prevents RB phosphorylation and leads to a decrease in E2F activity. In prostate cancer, several clinical trials for BET and CDK4/6 inhibitors are ongoing or completed in mCRPC patients (55–57). However, NEPC patients with *RB1* mutations or loss-of-function would show limited response to these CDK4/6 inhibitors. Therefore, finding alternative treatment approaches to modulate the cell cycle are significant and thus, development of a ZBTB7A inhibitor is relevant. Altogether, our study demonstrated an oncogenic role of ZBTB7A in NEPC and provides a rationale for targeting ZBTB7A in NEPC patients.

## 4 Materials and methods

### 4.1 Dependency analysis

Gene dependency data is based on pooled genome-scale shRNA or CRISPR/Cas9 screens from DEMETER2 Data v6 (DEMETER2) (33) or DepMap Public 20Q4 v2 (CERES) data (34). DEMETER2 or CERES scores for ZBTB7A were plotted against the scores for RET and the Pearson correlation coefficient was computed in each group of cell lines to evaluate the linear relationship between the two genes. To further statistically compare the patterns of RET dependency to ZBTB7A in each group of cell lines, we ranked the DEMETER2 or CERES scores of cell lines in each group and computed the Spearman correlation coefficient for ZBTB7A dependency relative to RET dependency.

### 4.2 Gene network construction and analyses

Two gene network matrices were generated, one for ADCA (genes = 19,957) and one for NEPC (genes = 19,835), by performing correlations based on the relative association of all gene pairs on WTS data from 33 ADCA and 14 NEPC tumors (1). From these context specific gene networks, the gene networks of individual genes were extracted. The correlation coefficient between +1 to -1 of gene networks represent a measurement of similarity, where +1 represents similar behavior, 0 represents independent gene behavior, and -1 represents dissimilar gene behavior. To generate a gene network signature from *ZBTB7A* and *RET* in each cohort, only the gene networks with correlation coefficients above 0.7 were used. To determine the score of gene network similarity in the other state (ADCA vs NEPC), the same gene list was used and the correlation coefficients were extracted from the other gene network. This analysis was depicted using violin plots where similar gene networks have overlapping violins (positive score) and dissimilar gene networks have oppositional violins (negative score).

### 4.3 Gene set enrichment analysis (GSEA)

Two gene profiles of *ZBTB7A* were generated by deriving Pearson correlation coefficients of *ZBTB7A* with all other detected genes in CRPC samples based on the gene expression data of 33 ADCA and 14 NEPC tumors from *Beltran et al.* (1). The differences between the NEPC and ADCA correlation coefficient values were then used to construct the specific association of *ZBTB7A* with all gene transcripts in NEPC as compared to ADCA. GSEA (42) pre-ranked analyses was then performed on this profile to identify enrichment of gene signatures from the 50 hallmark and 187 C6 oncogenic signatures in MSigDB (43).

### 4.4 Cell culture

Human prostate cancer NCI-H660 cells were purchased from the ATCC, and cells were validated annually by Promega PowerPlex16HS Assay at the University of Arizona Genetics core. Mycoplasma contamination was tested in each cell line every three months by the polymerase chain reaction method. Cells were cultured in Advanced DMEM/F12 (Gibco) medium supplemented with 1× B27 Supplement (Gibco), 10 ng/mL EGF (PeproTech), 10 ng/mL bFGF (PeproTech), 1% penicillin–streptomycin and 1× Glutamax (Life Technologies). NCI-H660 shZBTB7A cell lines were maintained in the medium containing 0.5 μg/mL puromycin.

### 4.5 Generation of stable *ZBTB7A* knockdown cell lines

Two pLKO.1 –shZBTB7A plasmids (ZBTB7A-1: CCACTGAGACACAAACCTATT, ZBTB7A-2: GCTGGACCTTGTAGATCAAAT) were used to stably knockdown *ZBTB7A* expression in NCI-H660 cells. The plasmids were selected from the RNAi Consortium shRNA library and purchased from MilliporeSigma (MISSION^®^ TRC shRNA TRCN0000136851, TRCN0000137332). pLKO.1 scramble shRNA plasmid was a gift from David Sabatini (Addgene plasmid #1864; http://n2t.net/addgene:1864; RRID : Addgene_1864) (58). To generate lentiviral particles, 293T cells were transfected with 6 μg pMDL, 3 μg pRev, 0.9 μg pVSVg, and 9 μg pLKO.1 shRNA plasmids using TransIT-LT1 (Mirus Bio). Then, NCI-H660 cells were transduced with lentivirus in media containing 8 μg/mL polybrene (Sigma-Aldrich). After 72 hours of infection, stable cells were selected by 0.5 μg/mL puromycin.

### 4.6 Western blot analysis

Cells were lysed in 10 mM Tris-HCl pH 8.0, 1 mM EDTA, 1% Triton-X 100, 0.1% Na deoxycholate, 0.1% SDS, 140 mM NaCl, and freshly supplemented protease (Roche) and phosphatase inhibitors (Thermo Scientific). The protein concentration was determined using Pierce BCA protein assay kit according to the manufacturer’s protocol. Cell lysates were boiled with 5× sample loading buffer at 95°C for 5 minutes prior to the analysis. Equal amount of protein (15 – 30 μg) from each cell lysate was loaded into Bio-Rad 4–20% Mini-PROTEAN TGX Stain-Free protein gel, transferred to a polyvinylidene difluoride membrane, blocked in 5% BSA in 1× TBS for 30 minutes at room temperature and incubated overnight at 4°C with primary antibodies diluted in 1% BSA in TBST. Membranes were washed three times with 1× TBST, incubated with LI-COR IR-conjugated secondary antibodies (1:5,000 dilution) for 1 hour at room temperature, washed again with 1× TBST and scanned using the Bio-Rad ChemiDoc MP Imaging System. Bio-Rad Image Lab 6.0 software was used to adjust images. The following primary antibodies were used at 1:1,000 dilution unless otherwise indicated: ZBTB7A (Cell Signaling Technology D7U2O), RET (Cell Signaling Technology E1N8X), p18 (Cell Signaling Technology DCS118), p21 (Cell Signaling Technology 12D1), p27 (Cell Signaling Technology D69C12), Bcl-2 (Cell Signaling Technology 124), Bax (Cell Signaling Technology 2D2), β-actin (Cell Signaling Technology 13E5, 1:5,000) and GAPDH (Santa Cruz Biotechnology 6C5, 1:5000). β-actin and GAPDH were used as loading controls.

### 4.7 Cell proliferation assay

Cells were seeded into 96-well plates at cell density of 2,000 cells/well (*n* = 6). Then, cells were cultured for indicated length of days with replenishing cell culture media every 5 days. Cell proliferation was measured every 7 days using WST reagent (Takara) according to manufacturer instructions. For measuring areas of cell clusters, one image was taken at the center of each well (50× magnification) and area of 15 cell clusters were manually measured using Zen lite software (Carl Zeiss Microscopy). The representative images of area measurements are shown in **Supplementary Figure 3**. Three biological replicates of assay were performed to confirm the results.

### 4.8 Cell cycle analysis

Cells were synchronized by culturing in Advanced DMEM/F12 medium without B27 and growth factor supplements for 24 hours and then supplemented medium for 48 hours. On the day of harvest, cells were trypsinized to dissociate clusters and filtered through 35 μm nylon mesh before fixation with 70% ethanol overnight at -20 °C. Cells were then washed with PBS and incubated with RNase A (50 μg/mL; Sigma-Aldrich) in PBS for 30 minutes at room temperature followed by propidium iodide (50 μg/mL; Sigma-Aldrich) in PBS. Cellular DNA content was measured with a LSRII flow cytometer (BD Biosciences). The distribution of cells in each phase of the cell cycle was analyzed by FlowJo (TreeStar). 50,000 cells were used for each analysis. Three biological replicates of experiment were performed to conform the results. The relative reduction of S phase cell population in shZBTB7A cell lines to the shScr cell line was calculated as follows:


$$Relative\;\%\;S\;phase\;reduction=\left(1-\frac{Average\;of\;shZBTB7A\;\%\;S\;phase}{Average\;of\;shScr\;\%\;S\;phase}\right)\times \;100$$

### 4.9 siRNA transfection

RNA interference of *RET* was performed using 27-bp siRNA duplexes purchased from Integrated DNA Technologies (IDT; Design ID hs.Ri.RET.13.1 and hs.Ri.RET.13.2). Cells were plated in 6-well plates at cell density of 1 × 10^6^ cells/well in Advanced DMEM/F12 medium without B27 and growth factor supplements. The cells were transfected with 30 nM siRNA duplexes using Lipofectamine RNAiMAX (Invitrogen) according to the manufacturer’s protocol. After 24 hours, the media was changed to the medium containing B27 and growth factor supplements, and then the freshly prepared lipid-siRNA complexes were added for additional 72 hours of transfection. Non-targeting siRNA duplex (IDT; 51-01-14-04) was used as a negative control for direct comparison. At the end of the experiment, the cells were harvested for western blot analysis or cell cycle analysis.

### 4.10 Annexin V/7-amino-actinomycin D (AAD) staining

Apoptotic cells were quantified by Annexin V-FITC and 7-AAD double staining according to the manufacturer’s protocol (Biolegend). On the day of harvest, the cells were trypsinized to make cells into single cells. Then, 1 × 10^6^ cells were resuspended in 100 μl of 1× Annexin V binding buffer and 2 μl of each Annexin V-FITC and 7-AAD viability staining solution were added to the cell suspension. After 15 minutes incubation at room temperature in the dark, 350 μl of Annexin V binding buffer were added and the stained cells were analyzed by a LSRII flow cytometer. 30,000 cells were used for each analysis. Three biological replicates of experiment were performed to conform the results.

### 4.11 Statistical analysis

The data were presented as the mean ± SD for the indicated number of independently performed experiments. The statistical significance (*p*<0.05) was determined using GraphPad Prism 9 with the tests indicated in the figure legends. The statistical tests were selected after testing the normality of the data distribution by Shapiro-Wilk and Kolmogorov-Smirnov, and the assumption of homogeneity of variance by Brown-Forsythe Test using GraphPad Prism 9. Dunnett’s multiple comparisons test and Dunn’s multiple comparisons test were performed after one-way ANOVA and Kruskal-Wallis test, respectively, and the adjusted *p*-values are shown.

## Data availability statement

The original contributions presented in the study are included in the article/**Supplementary Material**. Further inquiries can be directed to the corresponding author.

## Author contributions

SYB and JMD conceived the project. SYB designed, conducted experiments and analysed data under supervision of JMD. HEB, AD, and JHH performed gene network analysis and provided project discussion. JTG helped with flow cytometry experiments and contributed to the result discussion with TSF. ZES, and GL performed experiments with LuCaP PDX tumors, and EC and SRP provided the PDX tumor resources. SYB wrote the manuscript draft with support from JMD. All authors provided constructive critiques in editing the manuscript. All authors contributed to the article and approved the submitted version.
