# Supplementary material for: ZBTB7A as a novel vulnerability in neuroendocrine prostate cancer
Source: Front Endocrinol (Lausanne). 2023 Mar 29;14:1093332. doi: 10.3389/fendo.2023.1093332 (PMC10090553; doi:10.3389/fendo.2023.1093332)
Supplement: Supplementary file 1 [file DataSheet_1.pdf]

Supplementary Figures

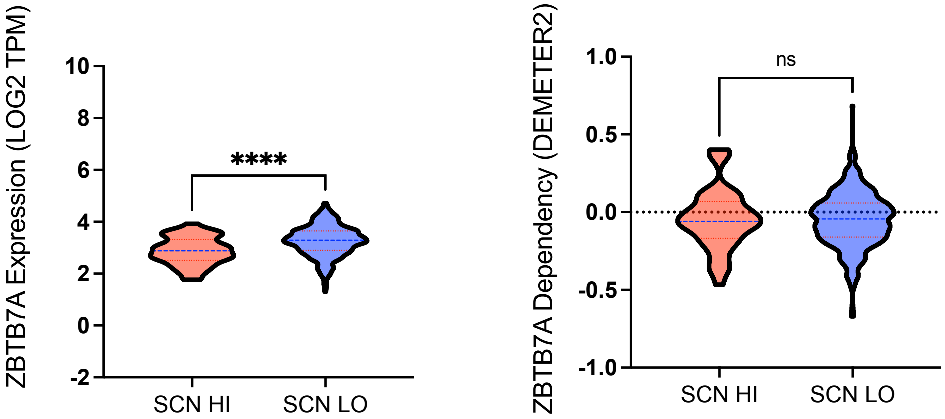

Supplementary Figure 1. ZBTB7A dependency scores alone is not sufficient to distinguish SCN HI and LO group of cell lines. (A) Comparing *ZBTB7A* mRNA expression in SCN HI versus SCN LO cell lines. *ZBTB7A* mRNA expression values were obtained from Cancer Cell Line Encyclopedia Cancer. ****p<0.0001 by Mann-Whitney test. (B) Comparing relative ZBTB7A dependency scores (DEMETER2) in SCN HI versus SCN LO cell lines. DEMETER2 scores were taken from DepMap (DEMETER2 Data v6). *ns*, non-significant by t-test.

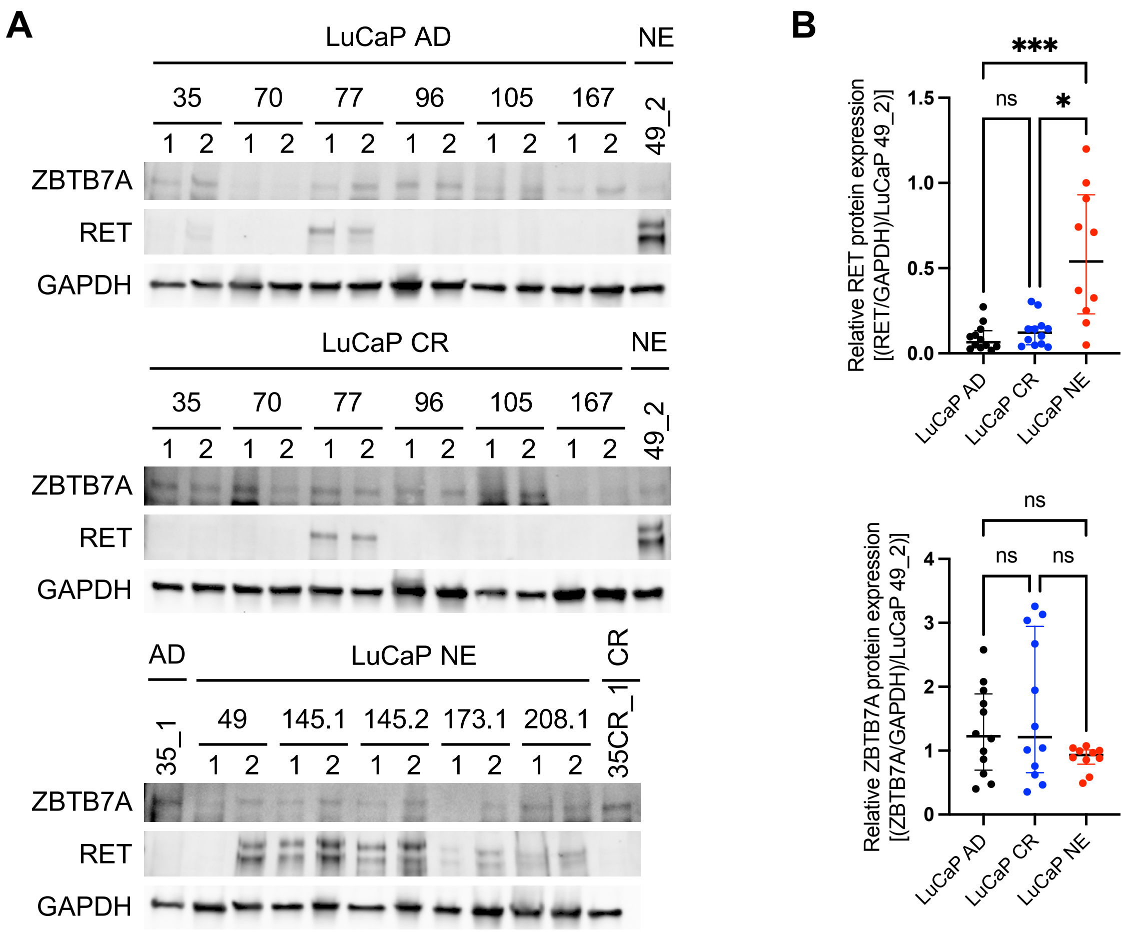

Supplementary Figure 2. RET and ZBTB7A are co-expressed in LuCaP neuroendocrine (NE) patient-derived xenograft (PDX) tumors. (A) Expression of RET and ZBTB7A proteins in LuCaP PDX tumors with adenocarcinoma (AD), castration resistant adenocarcinoma (CR) and neuroendocrine (NE) features. Two biological replicates of each LuCaP tumor model were analyzed and GAPDH was used as a loading control. (B) Protein expression levels were quantified by densitometry using Image J software. Each dot represents the mean from two technical replicates for each LuCaP model, and median and interquartile range are shown for each group. *ns*, non-significant, **p*<0.05, and ****p*<0.001 by Kruskal-Wallis test.

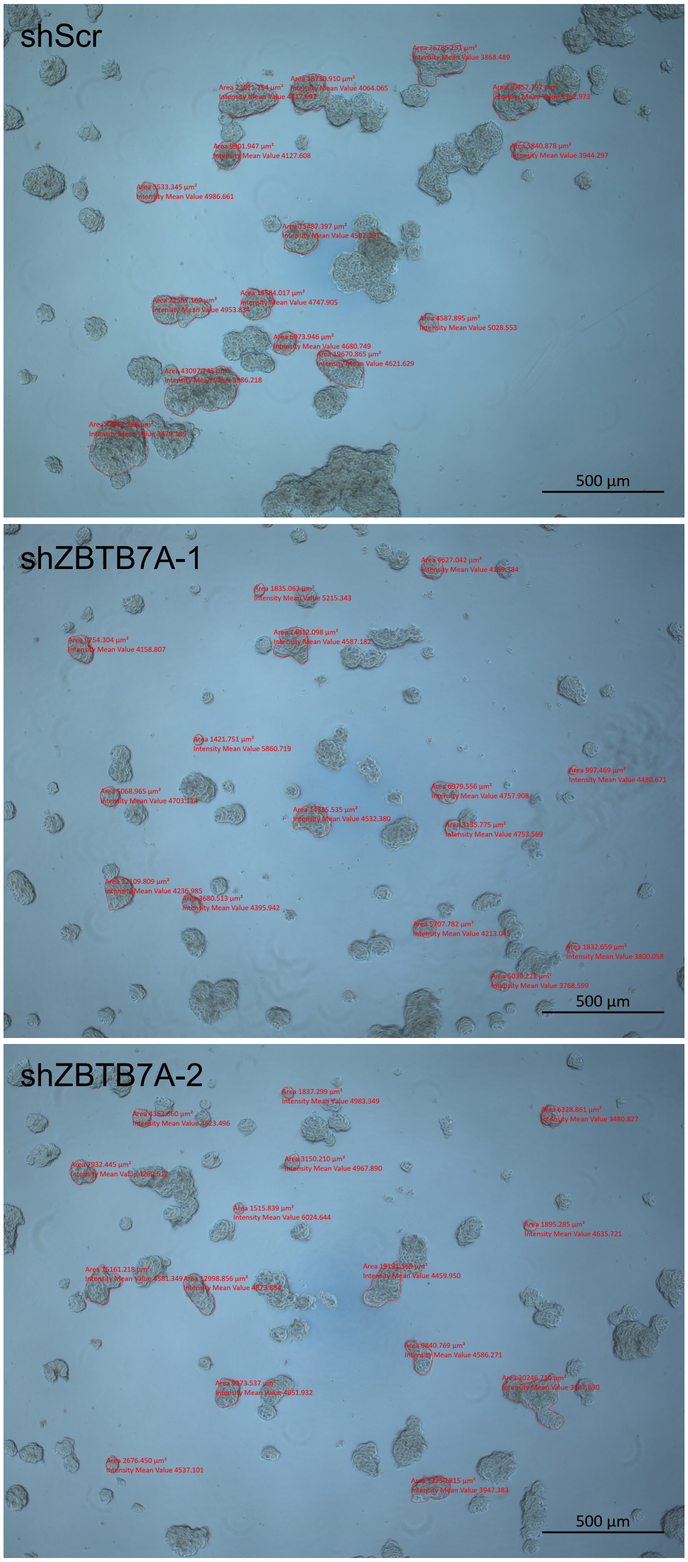

Supplementary Figure 3. Representative images of cell clusters manually measured for area using Zen lite software.
